# TLR4-mediated release of heparin-binding protein in human airways: a co-stimulatory role for IL-26

**DOI:** 10.3389/fimmu.2023.1178135

**Published:** 2023-05-10

**Authors:** Magnus Paulsson, Eduardo I. Cardenas, Karlhans F. Che, Bettina Brundin, Margaretha Smith, Ingemar Qvarfordt, Anders Lindén

**Affiliations:** ^1^ Division of Infection Medicine, Department of Clinical Sciences, Faculty of Medicine, Lund University, Lund, Sweden; ^2^ Department of Clinical Microbiology, Laboratory Medicine, Skåne University Hospital, Lund, Sweden; ^3^ Division of Lung and Airway Research, Institute of Environmental Medicine, Karolinska Institutet, Stockholm, Sweden; ^4^ Division of Respiratory Medicine and Allergology, Department of Internal Medicine and Clinical Nutrition, Sahlgrenska Academy, University of Gothenburg, Gothenburg, Sweden; ^5^ Karolinska Severe COPD Center, Department of Respiratory Medicine and Allergy, Karolinska University Hospital Solna, Stockholm, Sweden

**Keywords:** heparin-binding protein (HBP), lipopolysaccharide (LPS), toll-like receptor 4 (TLR4), IL-26, CAP37, azurocidin, neutrophils

## Abstract

**Background:**

Bacterial infection causes accumulation of neutrophils that release antimicrobial proteins including heparin-binding protein (HBP). In human airways, this neutrophil accumulation can be re-capitulated via intrabronchial exposure to lipopolysaccharide (LPS), a Toll-like receptor 4 (TLR4) agonist, that also causes a local increase in the neutrophil-mobilizing cytokine IL-26. Although LPS is considered a weak stimulus for HBP release *ex vivo*, its effect on HBP release in human airways *in vivo* has not been characterized.

**Methods:**

We determined whether intrabronchial exposure to LPS causes concomitant release of HBP and IL-26 in human airways, and whether IL-26 can enhance LPS-induced release of HBP in isolated human neutrophils.

**Results:**

We found that the concentration of HBP was markedly increased in bronchoalveolar lavage (BAL) fluid 12, 24, and 48 hours after LPS exposure, and that it displayed a strong and positive correlation with that of IL-26. Moreover, the concentration of HBP in conditioned media from isolated neutrophils was enhanced only after co-stimulation with LPS and IL-26.

**Conclusions:**

Taken together, our findings indicate that TLR4 stimulation causes concomitant release of HBP and IL-26 in human airways, and that IL-26 may constitute a required co-stimulant for HBP release in neutrophils, thus enabling the concerted action of HBP and IL-26 in local host defense.

## 1 Introduction

Neutrophils are key effector cells in airway host defense against pathogenic bacteria, as evidenced by the fact that neutropenic subjects (1) and neutrophil-depleted mice (2–4) are highly susceptible to severe pulmonary infections. Although neutrophils are virtually absent in healthy human airways, they are rapidly recruited from surrounding capillaries upon infection (5) and can subsequently eliminate invading bacteria via phagocytosis, formation of extracellular traps (NETs), and release of antimicrobial proteins such as heparin-binding protein (HBP).

The multifunctional protein HBP, also known as azurocidin or CAP37, is released from the azurophilic granules and secretory vesicles of activated neutrophils (6). Besides its potent antimicrobial activity against Gram-negative bacteria (7), HBP can also activate monocytes (8, 9) and mediate monocyte (10–12) and T-cell (13) recruitment. In addition, HBP participates in the increase in vessel wall permeability that is necessary for neutrophil extravasation (14). Finally, the concentration of HBP in bronchoalveolar lavage (BAL) fluid may constitute a biomarker for ventilator-associated pneumonia (15) and pulmonary infection in patients receiving lung allografts (16).

To characterize the mobilization of neutrophils caused by bacterial airway infection in humans, we and others have utilized intrabronchial exposure to lipopolysaccharide (LPS) in healthy volunteers as a model (17–21). This model is relevant to bacterial infection, given that LPS is a major component of Gram-negative bacteria that triggers the release of neutrophil-mobilizing cytokines from structural and immune cells of the lung via the Toll-like receptor 4 (TLR4) (17). Previous studies have shown that intrabronchial exposure to LPS causes rapid accumulation of neutrophils that resolves after approximately 48 hours (h) (18). In addition, we have previously utilized this model to demonstrate that LPS exposure elicits the release of cytokines associated with T helper 17 (Th17) cells in the airways (20), including IL-26 (21, 22).

The cytokine IL-26 is abundantly expressed in healthy human airways during stable conditions (21, 23), and its expression is enhanced during bacterial infection (24), whereupon IL-26 can contribute to airway host defense via its neutrophil-mobilizing and antibacterial properties. Notably, neutrophils express the IL-26 receptor complex (IL-10R2/IL-20R1) (21), and stimulation of human neutrophils with IL-26 *ex vivo* enhances their chemotactic response to IL-8 and fMLP, an archetype chemokine and a pathogenically relevant bacterial component, respectively (21), while at the same time inhibiting the non-specific release of neutrophil elastase and myeloperoxidase, two potentially self-damaging enzymes (24). In addition, we have demonstrated the involvement of IL-26 in human bacterial pneumonia (24) and provided confirming evidence that IL-26 exerts direct antibacterial effects, resembling those of an antimicrobial peptide, thereby forwarding IL-26 as a kinocidin (24–26).

For the current study, we hypothesized that local exposure to LPS induces the release of HBP in human airways and predicted that co-stimulation with at least one more molecule present in human airways (e.g., IL-26) is required for this release, given that LPS alone is a weak inducer of HBP release in isolated neutrophils (27–29). Thus, we first determined whether local exposure to LPS triggers the concomitant release of HBP and IL-26 in human airways. Then, we established whether co-stimulation with LPS and IL-26 can induce the release of HBP in isolated neutrophils. Finally, we determined whether the local release of HBP in human airways correlates with the local accumulation of neutrophils, macrophages or lymphocytes.

## 2 Materials and methods

### 2.1 BAL samples from healthy volunteers exposed to LPS and vehicle intrabronchially

A detailed description of the current study material has previously been published (19, 20). In brief, healthy volunteers were recruited at the Division of Respiratory Medicine, Sahlgrenska University Hospital, Gothenburg, Sweden. The study consisted of a first clinical visit for medical examination and screening, and two subsequent bronchoscopies for volunteers who matched the inclusion criteria. All included volunteers were never-smokers, non-atopic, without any regular medication other than oral contraceptives, no history of infection in the four weeks prior to bronchoscopy, had a normal ventilatory lung capacity (including forced expiratory volume in 1 s [FEV1] > 80% of predicted), as well as normal findings in their respective electrocardiogram and physical examination. During the first bronchoscopy, 10 ml of a PBS solution containing LPS (4 ng/kg in PBS; from *Escherichia coli* 0113:H10) or vehicle (PBS) was instilled in contralateral bronchial segments of healthy volunteers, thus allowing for matched intervention and control sampling within each subject. During the second bronchoscopy, BAL fluid samples were harvested from the contralateral LPS- and vehicle-exposed bronchial segments 12, 24, or 48 h after the first bronchoscopy. Cell-free BAL fluid was obtained by centrifugation (400 RCF, 10 min, 4°C), and cell pellets were re-suspended in PBS and used to determine total and differential cell counts using a hemocytometer and modified Wright-Giemsa-stained cytospins, respectively. In total, 32 healthy volunteers were included in the current study of which 25 completed all parts required for the current analysis.

In accordance with the Helsinki Declaration, all participants provided oral and written informed consent, after the ethical review of our protocol was approved by the Regional Ethical Review Board in Gothenburg (Diary No. 618-02; T065-04 and T638-07).

### 2.2 Isolation and stimulation of human neutrophils

Whole blood from human healthy donors was obtained at the Blood Donor Center (Karolinska University Hospital, Solna) and neutrophils were isolated using the MACSxpress Whole Blood Neutrophil Isolation Kit (Miltenyi Biotec, Bergisch Gladbach, Germany). In brief, 8 ml of whole blood per donor were incubated (5 min, room temperature) with antibody-conjugated magnetic beads that recognize surface antigens on all cell types present in human blood except neutrophils (negative selection). Samples were then subjected to a magnetic field (15 min) and non-target cells bound to the tube walls. Unbound neutrophils in plasma were pipetted into a separate tube and residual erythrocytes were removed via magnetic separation as described above using the MACSxpress Erythrocyte Depletion Kit (Miltenyi Biotec). Neutrophils were separated from plasma by centrifugation (400 RCF, 5 min), and resuspended in RPMI-1640 containing 0.2% penicillin-streptomycin (both from Gibco, Waltham, MA) and 10% donor-specific plasma. Resuspended neutrophils were seeded in 24-well plates (1×10^6^ cells/well) and stimulated (6 or 18 h, 37°C, 5% CO_2_) in duplicate with LPS (*E. coli* 0127:B8, Sigma-Aldrich, Burlington, MA), recombinant human IL-26 dimer protein, human anti-IL-26 antibody, IgG isotype control (all from R&D Systems, Minneapolis, MN), or PBS (control).

### 2.3 Quantification of HBP and IL-26

Protein concentrations of HBP and IL-26 in cell-free BAL fluid and conditioned media were quantified using enzyme-linked immune sorbent assay (ELISA; HBP: Axis-Shield Diagnostics, Dundee, United Kingdom; IL-26: Cusabio, Wuhan, China) in accordance with the manufacturers’ instructions. The individual IL-26 concentrations in cell-free BAL fluid were also utilized in a previous publication, although in a different scientific context (21). Moreover, a preliminary account of the data on HBP concentrations in cell-free BAL fluid was previously presented in abstract form (30). The *ex vivo* data, as well as the specific comparisons presented in the current study have not been published elsewhere.

### 2.4 Statistical analysis

Comparisons between two groups were conducted by paired Student’s t-test following log_10_-transformation of the original data. Multiple comparisons were performed by non-parametric Friedman’s test followed by Dunn’s *post hoc* test to compare each group against the control. Correlations were determined by Spearman’s rank test. All statistical analyses were carried out in Prism 9.4.0 (GraphPad, San Diego, CA).

## 3 Results

### 3.1 LPS exposure induces the concomitant release of HBP and IL-26 in human airways

**Figure 1:**
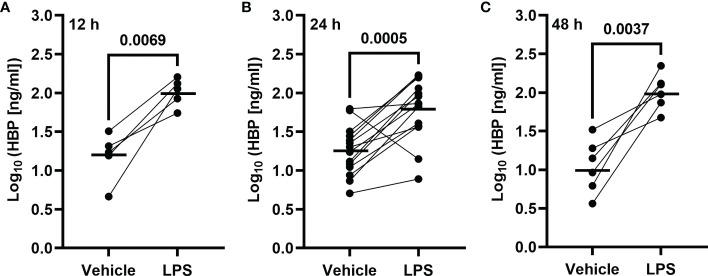
Intrabronchial exposure to LPS *in vivo* and protein concentrations of HBP in BAL samples from healthy volunteers. Concentrations of HBP quantified in cell-free BAL fluid from healthy volunteers harvested **(A)** 12 (n = 5), **(B)** 24 (n = 14), or **(C)** 48 (n = 6) h after contralateral bronchial segment exposure to LPS and vehicle (PBS), respectively. Circles joined by a line represent individual subjects. Mean values are indicated by bold horizonal lines. Statistical significance was determined by paired Student’s t-test after log_10_-transformation.

**Figure 2:**
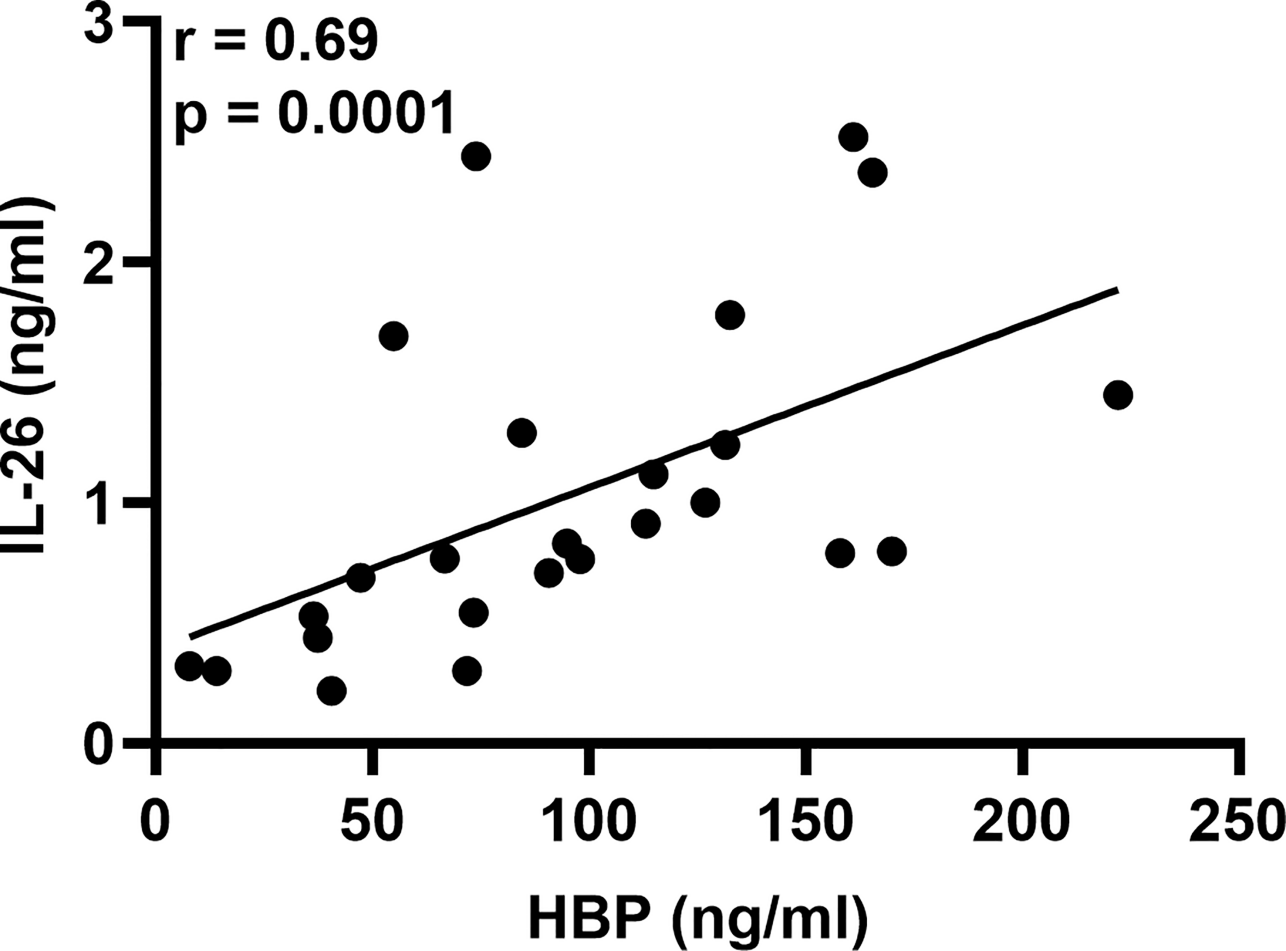
Association of HBP and IL-26 concentrations in BAL samples from healthy volunteers after intrabronchial exposure to LPS *in vivo.* Spearman correlation analysis of the protein concentrations of HBP and IL-26 quantified in cell-free BAL fluid from LPS-exposed bronchial segments. Closed circles represent individuals from the 12, 24, and 48 h time points.

To determine whether LPS could trigger the concomitant release of HBP and IL-26 in human airways, we harvested BAL fluid 12, 24, and 48 h after LPS instillation. We found that the protein concentrations of HBP were markedly increased in cell-free BAL fluid from LPS-exposed bronchial segments at all time points (**Figures 1A–C**). Notably, the protein concentrations of HBP in BAL fluid from LPS-exposed bronchial segments did not display any statistically significant difference across time points (ANOVA: p-value = 0.26). Moreover, we have previously used this study material to establish that LPS exposure elicits a substantial release of IL-26 in human airways (21). Here, we found that the protein concentrations of HBP in BAL fluid from the LPS-exposed bronchial segments displayed a strong and positive correlation with the corresponding concentrations of IL-26 (**Figure 2**).

### 3.2 Co-stimulation with LPS and IL-26 induces the release of HBP in human neutrophils

**Figure 3:**
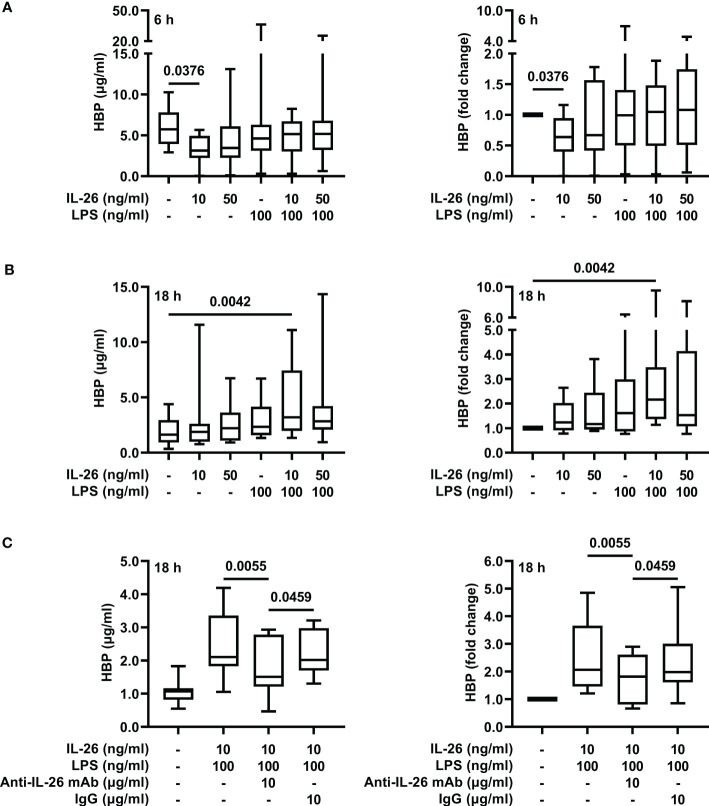
Co-stimulation with IL-26 and LPS increases the concentrations of HBP in conditioned media from human neutrophils *ex vivo*. Blood neutrophils were isolated from 8 **(A, B)** or 10 **(C)** healthy donors and stimulated with LPS and/or IL-26 alone, or in the presence of a blocking anti-IL-26 antibody or its isotype control, for **(A)** 6 or **(B, C)** 18 h. The protein concentrations of HBP were quantified by ELISA in cell-free conditioned media. Data is presented as median with range of protein concentrations (left) and fold-change of each condition compared to unstimulated neutrophils (right). Statistical significance was determined by non-parametric Friedman’s test followed by Dunn’s *post hoc* test to compare each group against the control **(A, B)** or the blocking antibody group **(C)**.

To determine whether co-stimulation with IL-26 can induce the release of HBP in LPS-exposed neutrophils, we stimulated blood neutrophils with IL-26 and LPS alone or in combination, and measured HBP concentrations in cell-free conditioned media 6 and 18 h later. Although stimulation with IL-26 alone caused a reduction in the concentrations of HBP at the 6 h time point (**Figure 3A**), we found that stimulation with LPS alone could not induce a significant increase in HBP concentrations (**Figures 3A, B**). However, co-stimulation of blood neutrophils with IL-26 and LPS for 18 h resulted in an average 3-fold increase in HBP concentrations compared to unstimulated neutrophils (**Figure 3B**). To confirm these findings, we stimulated blood neutrophils with IL-26 and LPS in the presence of a blocking anti-IL-26 antibody or an isotype control and found that incubation with the blocking antibody inhibited the IL-26- and LPS-dependent increase in HBP concentrations (**Figure 3C**).

### 3.3 The concentrations of HBP and leukocytes in BAL fluid correlate after LPS exposure

**Figure 4:**
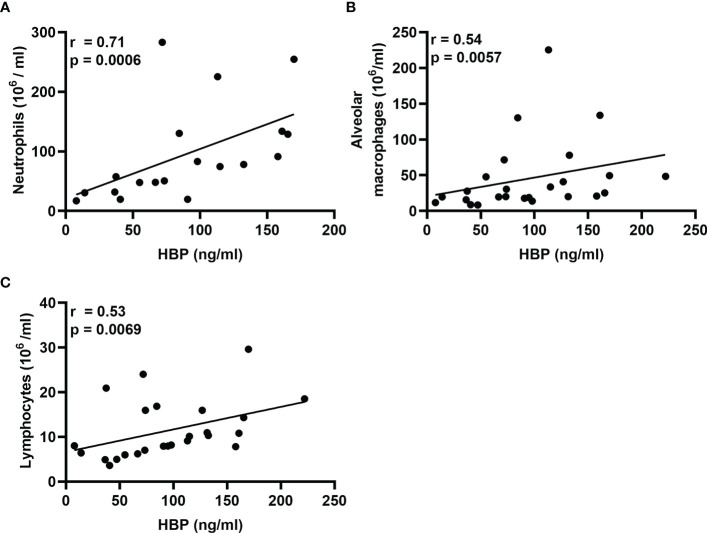
Associations of HBP and leukocytes in BAL samples from healthy volunteers after intrabronchial exposure to LPS *in vivo*. Spearman correlation analyses of the protein concentrations of HBP quantified in BAL samples from LPS-exposed bronchial segments and corresponding concentrations of **(A)** neutrophils, **(B)** alveolar macrophages, and **(C)** lymphocytes. Closed circles in **(A)** represent individuals from the 12 and 24 h time points only, and in **(B, C)** represent individuals from the 12, 24, and 48 h time points.

In a previous publication on this study material (20), we showed that intrabronchial exposure to LPS causes a rapid increase in the local concentrations of neutrophils, alveolar macrophages, and lymphocytes. Notably, we have also shown that this increase in the concentrations of neutrophils begins to resolve 48 h after exposure, while the concentrations of alveolar macrophages and lymphocytes remain high even at this time point (19). To date, neutrophils are the only verified source of HBP, and we now found that the protein concentrations of HBP in BAL fluid displayed a strong correlation with those of neutrophils during the first 24 h after LPS exposure (**Figure 4A**). Moreover, previous publications have demonstrated that HBP is a chemoattractant for monocytes (10) and T-cells (13) *ex vivo*, and we now found that the protein concentrations of HBP in BAL fluid of LPS-exposed bronchial segments correlated with those of alveolar macrophages and lymphocytes (**Figures 4B, C**).

## 4 Discussion

In the present study, we show for the first time that intrabronchial exposure to LPS *in vivo* causes a substantial increase in HBP concentrations together with a pronounced accumulation of neutrophils in BAL samples from healthy volunteers. Moreover, we establish that this increase in HBP concentrations occurs rapidly and remains stable for 48 h after exposure. Importantly, we also show that the LPS-induced increase in HBP concentrations is associated with a concomitant increase in IL-26 concentrations.

Notably, our observation that LPS exposure causes an increase in HBP concentrations *in vivo* is in sharp contrast with previous studies on isolated neutrophils, in which *ex vivo* exposure to LPS failed to induce the release of HBP (27–29). Given our current results, it seems likely that co-stimulation with a molecule present in human airways—but absent in previous *ex vivo* experiments—is required to induce the release of HBP in LPS-exposed neutrophils. Moreover, it was recently shown that co-stimulation with certain pro-inflammatory cytokines such as TNFα and GM-CSF can enhance the LPS-induced activation and degranulation of neutrophils (31). Thus, we now demonstrate that co-stimulation with IL-26, which is abundant in naïve human airways, and even more so after LPS exposure, can induce the release of HBP in LPS-exposed neutrophils *ex vivo*.

Given that we previously forwarded evidence indicating that neutrophils release IL-26 in response to LPS (24), it may seem paradoxical that we now had to add exogenous IL-26 to induce the release of HBP in LPS-exposed neutrophils. However, we think that neutrophils encounter IL-26 from a wide range of cellular sources *in vivo*, and that this exogenous IL-26 is required to induce the LPS-mediated release of HBP. Indeed, the results from our previous work on IL-26 shows that several structural and immune cells found in human airways, such as primary lung fibroblasts (32), bronchial epithelial cells (preliminary data) (33), and alveolar macrophages (21), release IL-26 in response to LPS.

Neutrophils store pre-made HBP in two distinct subcellular compartments: azurophilic granules and secretory vesicles (6). Interestingly, multiple studies suggest that exocytosis of azurophilic granules can occur without HBP release. For instance, stimulation of neutrophils with LPS *in vitro* is known to cause the release of proteins found exclusively in azurophilic granules such as elastase or myeloperoxidase (MPO) (24, 31), but it does not induce a significant release of HBP (27–29). In line with this, we previously showed that stimulation of isolated neutrophils with LPS alone causes the release of MPO (24) and, using the same experimental conditions, we now show that LPS alone is not enough to trigger a significant release of HBP. Moreover, we also showed previously that co-stimulation with LPS and IL-26 has no additive effect on the release of MPO (24), and we now use the same experimental conditions to show that co-stimulation with LPS and IL-26 induces the release of HBP. Taken together, these findings suggest that the secretion pattern of HBP differs from those of other proteins found in azurophilic granules upon stimulation with LPS. Further research is necessary to elucidate the mechanisms behind these differences.

Notably, we have previously reported that stimulation of neutrophils with IL-26 alone for 6 h inhibits the release of elastase and MPO (24), and we now show that IL-26 can also inhibit HBP release at this (early) time point. However, this inhibitory effect is likely to be transitory, as evidenced by the fact that stimulation with IL-26 alone does not inhibit the release of HBP at a later time point.

Although neutrophils are the only consistently verified source of HBP in humans (6), LPS exposure has been shown to induce the release of HBP in the monocytic leukemia-derived cell line Mono Mac 6 (34). However, this finding has not been confirmed in human primary monocytes. Instead, studies on primary monocytes isolated from healthy subjects show that monocytes rapidly internalize exogenous HBP within 30 min, whereupon HBP can activate monocytes directly or amplify the stimulating effect of LPS (8, 35). Because the leukocyte population recruited by intrabronchial exposure to LPS was predominantly composed of neutrophils, and because the concentration of HBP in BAL fluid had the strongest correlation with that of neutrophils, it seems likely that neutrophils constitute the main source of HBP in our current human infection model. Nevertheless, future research is needed to determine whether monocytes are indeed capable of producing HBP in response to LPS or other bacterial stimuli. Finally, the fact that the concentrations of alveolar macrophages and lymphocytes in BAL fluid from LPS-exposed bronchial segments displayed positive correlations with those of HBP can be attributed to the proven capacity of HBP to act as a chemoattractant for monocytes (10–12) and lymphocytes (13).

In summary, the results from this interventional study on human subjects indicate that there is accumulation of neutrophils and concomitant release of HBP and IL-26 in airways exposed to an important component of Gram-negative bacteria. The results also indicate that IL-26 may constitute a required co-stimulant for this type of HBP release in neutrophils. Thus, HBP and IL-26 may act in concert to maintain local host defense against Gram-negative bacteria. Our findings motivate new studies to determine whether this co-stimulatory mechanism can be utilized as target for diagnosis or therapy during bacterial airway infection or pneumonia.

## Data availability statement

The original contributions presented in the study are included in the article/supplementary material. Further inquiries can be directed to the corresponding author.

## Ethics statement

The studies involving human participants were reviewed and approved by the Regional Ethical Review Board in Gothenburg (Diary No. 618-02; T065-04 and T638-07). The patients/participants provided their written informed consent to participate in this study.

## Author contributions

The study was conceived by MP, EC and AL. Clinical investigations were performed by MS and IQ, under the supervision of AL. Laboratory experiments and analyses were performed by MP, EC, KC in collaboration with BB, under the supervision of AL. The data interpretation was performed jointly by MP, EC. and AL, who also outlined the manuscript together. All authors contributed to the article and approved the submitted version.
